# Three-Dimensional Hepatocellular Carcinoma/Fibroblast Model on a Nanofibrous Membrane Mimics Tumor Cell Phenotypic Changes and Anticancer Drug Resistance

**DOI:** 10.3390/nano8020064

**Published:** 2018-01-25

**Authors:** Binh Duong Le, Donggu Kang, Seokhwan Yun, Young Hun Jeong, Jong-Young Kwak, Sik Yoon, Songwan Jin

**Affiliations:** 1 Department of Advanced Convergence Technology, Korea Polytechnic Univsersity, Siheung-si 15073, Gyoenggi-do, Korea; duonglb2468@gmail.com; 2 Department of Mechanical System Engineering, Korea Polytechnic Univsersity, Siheung-si 15073, Gyoenggi-do, Korea; kdgplant@naver.com; 3 Department of Mechanical Engineering, Korea Polytechnic Univsersity, Siheung-si 15073, Gyoenggi-do, Korea; yuntobi@kpu.ac.kr; 4 School of Mechanical Engineering, Kyungpook National University, Buk-gu, Daegu 702-701, Korea; yhjeong@knu.ac.kr; 5 Department of Pharmacology, Ajou University School of Medicine, Suwon 442-721, Korea; jykwak@ajou.ac.kr; 6 Department of Anatomy, Pusan National University School of Medicine, Yangsan 626-770, Korea; sikyoon@pusan.ac.kr

**Keywords:** tumor microenvironment, hepatocellular carcinoma, nanofibrous membrane

## Abstract

Three-dimensional (3D) in vitro tissue or organ models can effectively mimic the complex microenvironment of many types of human tissues for medical applications. Unfortunately, development of 3D cancer models, which involve cancer/stromal cells in a 3D environment, has remained elusive due to the extreme complexity of the tumor microenvironment (TME) and the stepwise progression of human cancer. Here, we developed hepatocellular carcinoma (HCC) models, which consist of fibroblasts as stromal cells, HCC cells, and a nanofibrous membrane to mimic the complex TME. The 3D HCC models were fabricated using three distinct culture methods: cancer cells grown directly on the nanofibrous membrane (mono model), fibroblasts covering the nanofibrous membrane (layer model), and both cancer cells and fibroblasts grown on the nanofibrous membrane (mixed model). Interestingly, the mono model and layer model showed similar tissue structures, whereas the mixed model resulted in phenotypic changes to the cancer cells. Further analysis demonstrated that the mixed models promoted the expression of fibronectin and vimentin, and showed higher resistance to anticancer drugs compared with the other models. Thus, our 3D HCC model could be utilized for testing efficient anticancer therapies at various stages of cancer, with potential application to different tumor types.

## 1. Introduction

Hepatocellular carcinoma (HCC) is the most common type of primary liver tumor, and represents the third leading cause of cancer-related deaths worldwide as well as the sixth most prevalent cancer overall [1]. In general, the tumor microenvironment (TME) is in constant flux due to tissue remodeling and changes in the recruitment of stromal cells, including a diverse array of immune cells that promote angiogenesis [2]. As the tumor increases in size, the cancer cells transform to a more aggressive phenotype through epithelial-to-mesenchymal transition (EMT), which is further accompanied by alterations in the TME of the extracellular matrix (ECM) [3,4]. In particular, fibroblasts, components of the tumor stromal cells, play an important role in tumor progression, growth, and metastasis, and recent research has begun to reveal their structural and functional contributions to these processes [5]. For these reasons, developing functional and gradational three-dimensional (3D) tumor models that can effectively mimic the TME and stromal cell effects is an essential goal for effectively testing drugs and investigating the mechanisms of tumor progression such as cancer metastasis and EMT.

It is well known that transforming growth factor-beta (TGF-β) is a potent inducer of EMT [6,7,8]. Thus, EMT can be induced by adding TGF-β to epithelial cells in two-dimensional (2D) culture [9] as well as in 3D culture [10,11,12]. However, TGF-β mediates tumor suppressor activity in a variety of cancers, including colon cancer, and cannot initiate EMT in retinal pigment epithelial cells [13]. Despite efforts to create a cancer model on a 2D substrate, a 2D cancer model (i.e., a monolayer culture model) cannot provide useful information on the drug responses influenced by the TME [14]. Although 2D cancer models are invaluable tools for identifying potential anticancer agents in the early stages of drug discovery, they have several disadvantages, including the rapid loss of cell function, difference of dimensionality from in vivo conditions, and difficulties of heterogeneous culture. Moreover, numerous studies have demonstrated that 3D cancer homospheroids show greater drug resistance than 2D cancer models, especially those consisting of cancer/stromal cells and hydrogels [15,16,17,18]. Therefore, the persistence of three dimensionalities and heterogeneous culture conditions may play an important role in engineering useful in vitro models for testing anticancer therapeutics.

To date, the failure rate of anticancer drugs in clinical trials has been extremely high at approximately 96% [19]. To improve the success rate and development of more effective drugs that best represent the actual cancer environment, current in vitro models used in pre-clinical tests should be supplemented with various 3D culture methods (e.g., hydrogel-based spheroid culture or printing approaches, scaffold-based or scaffold-free culture, microfluidics).

A non-woven nanofibrous membrane has recently emerged as a promising candidate to mimic the true ECM structure. Owing to the randomly distributed nano-porous property and high surface-to-volume ratio, a non-woven nanofibrous membrane provides a 3D microenvironment for cell growth that is similar to the in vivo condition [20]. In contrast, a woven nanofibrous membrane has an isotropic structure that enables cells to align in the direction of the fibers; such membranes have been shown to promote the alignment of breast cancer cells and EMT [10]. In reality, epithelial clusters reside in a heterogeneous microenvironment whose mechanical properties vary not only in terms of stiffness but also topography, dimensionality, and confinement [21]. Interestingly, McLane et al. [11] demonstrated that use of a woven nanofiber alone was not sufficient to induce an EMT-like set of changes in breast epithelial cells, suggesting that that other factors such as paracrine/juxtacrine signaling and differential ECM ligand engagement facilitate the transition to a mesenchymal phenotype. Therefore, cancer/stromal cells, appropriate chemical reagents, and the TME are all crucial factors that must be effectively mimicked for development of a suitable cancer cell model to test the stepwise processes contributing to the response of a variety of tumor cells and the TME, including carcinoma invasion, metastasis, and EMT, as well as chemoresistance in the face of treatment.

From these points of view, we hypothesized that co-culturing fibroblasts with cancer cells on a 3D membrane can be a more physiologically relevant method of achieving an in vivo-like cancer morphology and microenvironment. Toward this end, we engineered 3D HCC models using three distinct culture methods with different culturing times: (1) only HCC cells cultured on a nanofibrous membrane (mono model); (2) culturing of fibroblasts for the first three days until they reached confluence on the nanofibrous membrane, and then HCC cells were seeded on top (layered model); and (3) co-culturing HCC cells with fibroblasts simultaneously (mixed model). We performed albumin enzyme-linked immunosorbent assay (ELISA) and a collagen enzyme immunoassay (EIA) to verify whether the cancer and stromal cells were viable, and identified chemoresistance in each model. Moreover, an immunofluorescence assay was conducted to identify the differentiation of tumor progression, such as EMT, among the three distinct models. Overall, we found that the mixed model induced phenotypic cell changes and the expression of EMT-related factors, and showed higher resistance to anticancer drugs compared with the other models. Thus, our proposed 3D HCC models could be utilized for testing anticancer therapies.

## 2. Results

### 2.1. Experimental Design and Characteristics of the Nanofibrous Membrane

**Figure 1:**
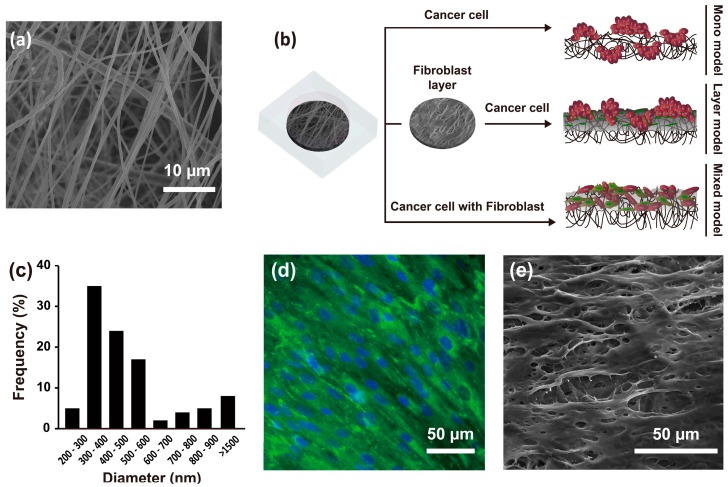
SEM image of the nanofibrous membrane (**a**) and schematic of the experimental design (**b**); Diameter distribution of the nanofibrous membrane (**c**) (*n* = 10); Fibroblast layer on a nanofibrous membrane after three days of culture with staining of collagen type I (green) and the nucleus (blue) (**d**); SEM image of the fibroblast layer on the nanofibrous membrane (**e**).

The average nanofibrous membrane diameter was 517 ± 32 nm (range, 200–1500 nm), with a peak diameter frequency of 300–600 nm (Figure 1a,c). The mean thickness of nanofibrous membrane was 70 ± 10 µm, mean pore area was 67 ± 46.81 µm, and percent porosity was 0.45. As described above, three different culture methods were applied to engineer the 3D HCC models. The mono model was made up of only Hep G2 liver HCC cells on the nanofibrous membrane. The layer model consisted of a fibroblast layer by pre-culturing CCD-1112Sk cells in advance for three days on the nanofibrous membrane, followed by overlapping of Hep G2 cells. The mixed model was a mixture of Hep G2 and CCD-1112Sk cells (ratio 1:1) cultured on the nanofibrous membrane, as shown in Figure 1b. In particular, the fibroblast layer used for developing the layer model, overlaid on the nanofibrous membrane, showed expression of collagen type I and topological characteristics as shown in Figure 1d,e.

### 2.2. Morphological Characteristics of Hep G2 Cells and Fibroblasts

**Figure 2:**
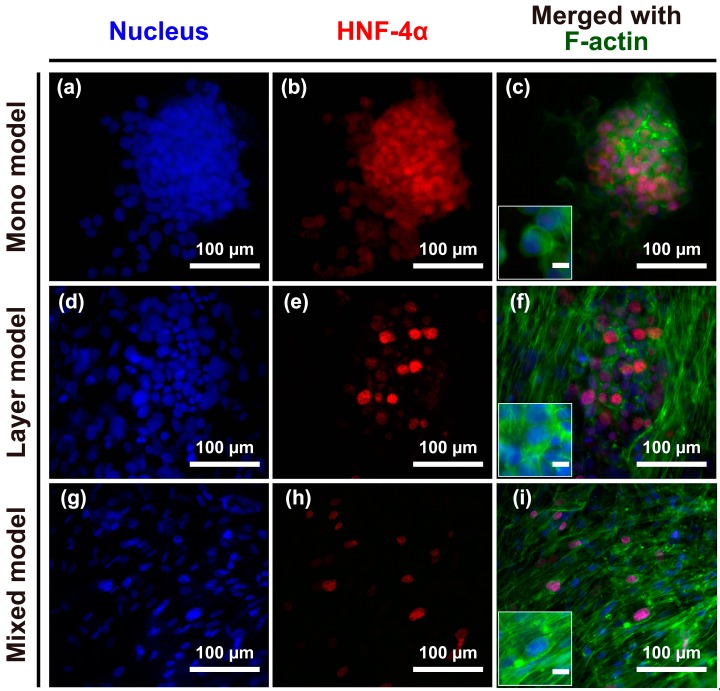
(**a**–**i**) Structural morphology of cancer cells and fibroblasts or cancer cells alone on the nanofibrous membrane after seven days of culture. Immunofluorescence of DAPI-stained nuclei (blue), hepatocyte nuclear factor 4 alpha (HNF4α, red), and the cytoskeleton protein F-actin (green). Merged images of the experimentally designed mono model (**c**); layer model (**f**); and mixed model (**i**). Unmarked scale bars, 10 μm.

The structural morphology of the engineered models was observed after seven days in culture. CCD-1112Sk and Hep G2 cells were counter-stained with DAPI (4′,6-diamidino-2-phenylindole) (Figure 2; blue) and phalloidin (Figure 2; green), and Hep G2 cells were further stained with hepatocyte nuclear factor-4 (HNF-4) (Figure 2; red) for batch-to-batch variable niche identification. Hep G2 cells were grown without the loss of cell-cell junctions on the nanofibrous membrane, as shown in the merged image in Figure 2c, mono model. There was no significant difference in the Hep G2 cell circularity between the mono and layer models, whereas cells in the mixed model were more elongated and spread with fibroblasts on the nanofibrous membrane. Thus, the structure of the nanofibrous membrane and the fibroblast layer containing the nanofibrous membrane could not effectively separate Hep G2 cells; however, a mixture of CCD-1112Sk and Hep G2 cells not only allowed for separation of the two cell types but could also elongate Hep G2 cells on the nanofibrous membrane.

### 2.3. Albumin and Collagen Secretion

**Figure 3:**
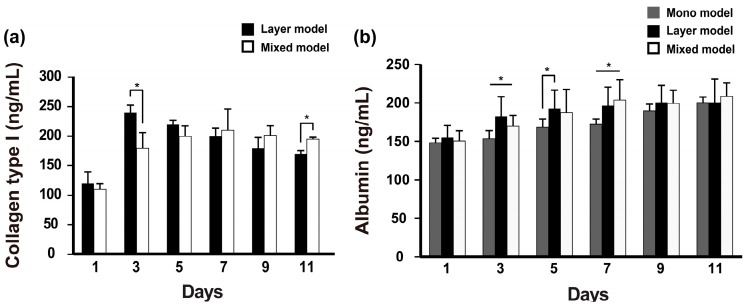
Albumin and collagen type I secretion from the experimentally designed models. (*n* = 3, * *p* < 0.05).

Fibroblast-secreted collagen was measured in the layer model and mixed model. The collagen levels in the layer model were higher than those in the mixed model on day 3, and then gradually decreased during cell culture. Although there was less secretion of collagen in the mixed model, the secreted collagen was stably retained over 11 days of culture (Figure 3a). Further, albumin secretion was measured on these scaffolds during 11 days. Unlike collagen secretion detected in our models, albumin secretion gradually increased over long-term culture and was detected at a higher level in the layer models on day 5 compared with the mixed model; however, this pattern reversed as of day 7 and thereafter (Figure 3b). The collagen secretion level in the mono model also gradually increased over the culture time and ultimately reached a similar level to that of the layer model on day 11. Thus, we confirmed that despite the different cell niches based on the seeding condition, both fibroblasts and Hep G2 cells co-existed on the nanofibrous membrane.

### 2.4. Cell-Cell Junctions in Hep G2 Cells

**Figure 4:**
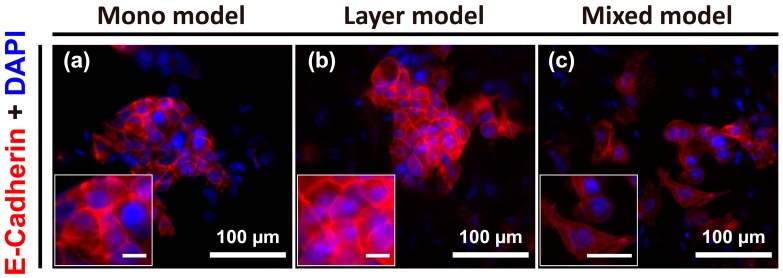
Images of E-cadherin expression at cell-cell junctions of Hep G2 cells under various culture conditions on day 7, which was not expressed in CCD-1112Sk fibroblasts, detected using E-cadherin antibody. Mono model (**a**); layer model (**b**); and mixed model (**c**). Unmarked scale bars, 10 μm.

To further identify the specific phenotypic changes in Hep G2 cells for the three distinct models, cell-cell junctions were visualized by E-cadherin staining. Reduction or loss of E-cadherin expression (i.e., cell-cell adhesion) is considerable markers of the first stage of EMT events. Otherwise, loss of cell-cell adhesion promotes metastasis by disaggregating cancer cells from one another and promoting a phenotypic change toward mesenchymal cells [22]. The result of this analysis showed clear E-cadherin expression at the cell-cell boundaries in both the mono model and layer model, and the Hep G2 cells were aggregated or vice versa (Figure 4a,b). However, the individual cell-to-cell distance in the mixed model was greater than that detected for the other two models, and E-cadherin expression was not clearly detected. Further, the morphology of the cells in the mixed model was more irregular or elongated compared to that of the cells in the other models (Figure 4c). Thus, the mixed model could be considered analogous to the mesenchymal state in various stages of tumor progression, unlike the mono model and layer model.

### 2.5. Vimentin and Fibronectin Expression

**Figure 5:**
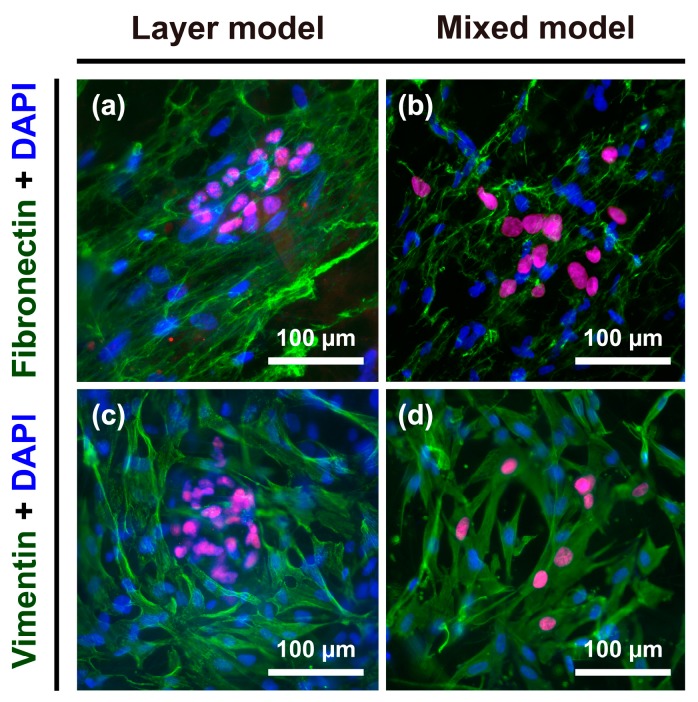
Immunofluorescence images of fibronectin and vimentin staining in Hep G2 cells and fibroblasts. Expression of fibronectin in the (**a**) layer model and (**b**) mixed model; expression of vimentin in the (**c**) layer model and (**d**) mixed model.

To further explore the phenotypic changes occurring in Hep G2 cells, the expression of fibronectin as an ECM protein, and vimentin as a cytoskeletal marker, in the progression of EMT was detected in the layer model and mixed model. Both fibronectin and vimentin are considered to be general mesenchymal makers acquired during EMT [23,24]. After seven days of culture, fibroblasts specifically showed strong expression of both fibronectin and vimentin (Figure 5), whereas Hep G2 cells were vaguely stained: fibronectin and vimentin were more greatly expressed in the mixed model (Figure 5b,d) than layer model (Figure 5a,c). 

### 2.6. Anticancer Drug Resistance

**Figure 6:**
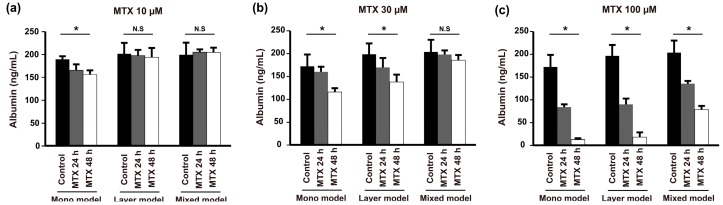
Anticancer drug resistance of Hep G2 cells determined in the mono model, layer model, and mixed model by measuring albumin secretion with increasing concentrations of MTX: (**a**) 10 µM; (**b**) 30 µM; and (**c**) 100 µM after 24 and 48 h. (*n* = 3, * *p* < 0.05, N.S = not significant).

Finally, to assess the effect of the various TMEs established with our models on anticancer drug resistance, we treated the models with different concentrations of methotrexate (MTX) and measured the level of albumin secretion from 24 to 48 h. Administration of a low dose of MTX (10 μM) resulted in different drug resistance behaviors between the mono model and layer model despite the fact that they showed similar morphological behavior (Figure 2c,f) and expression of E-cadherin in Hep G2 cells (Figure 4a,b). Low-dose MTX affected resistance of the mono model but not the layer model or mixed model (Figure 6a). However, the mono model and layer models showed similar susceptibility to 30 μM MTX, which was higher than that of the mixed model (Figure 6b). Furthermore, with high-dose MTX (100 μM), the albumin secetion of both the mono model and layer model was significantly reduced at 48 h, whereas the mixed model still showed anticancer drug resistance (Figure 6c). Taken together, these results indicate that these models show variation in the acquisition of drug resistance with various drug concentrations over time, and the mixed models treated with high-dose MTX showed higher drug resistance than the others. Thus, we confirmed that the drug response depends on the TME, which, in this case, is induced by heterogeneous cell locations, cell types, or scaffold.

## 3. Discussion

In most cases, cells are still cultured as 2D monolayers on flat surfaces, which does not accurately simulate the in vivo conditions such as cell-cell and cell-ECM interactions, and can even transform the cellular characteristics. To effectively mimic in vivo-like cellular activity, sophisticated 3D cancer models have been developed in recent years [25,26,27,28], and comparison of 2D and 3D cancer models have been useful for developing more effective methods for anticancer drug testing [27].

In general, 3D cancer models showed a higher resistance to the anti-cancer drug than 2D monolayers. 3D cancer model has an additional dimension with cell-cell and cell-ECM interactions which play pivotal role in diffusion of the drug to the cells. The results of our study showed that the mono model has cell-cell interaction with single-cell and cell-ECM, while the layer model and mixture model have both external surface of fibroblast and nanofibrous membrane. Thus, although administration of a low-dose of MTX to mono model is effective with cancer cells over time, there was no effect with the layer and mixed models (Figure 4a). However, high-dose drug-treatment decreased albumin secretion simultaneously in each model (Figure 4c). In particular, drug resistance in the mixed model was higher than the layer model, which is, we believe, caused by a phenotypical change of cancer cells.

Despite extensive efforts to engineer a 3D tumor model in hydrogels, the batch-to-batch variability, complex molecular composition, and uncontrolled degradation of hydrogels have limited the ability to study the influence of a particular property of the ECM on tumor cells while maintaining other variables unaltered. Compounding these difficulties, a scaffold fabricated with synthetic materials has no natural cell adhesion sites and is thus not readily remodeled by cells [29]. Given that collagen type I can induce EMT in some cell types through a distinct pathway independent of TGF-β [30], an ideal 3D tumor model would be one that allows various cells to be located in an appropriate niche (e.g., scaffolds and ECM materials) and distinguished to best recreate the various stages of tumor formation and progression. We hypothesized that a heterogeneous microenvironment composed of fibroblasts, cancer cells, and a nanofibrous membrane with different conditions—including topological characteristics, juxtacrine signaling, and fibroblast-secreted extracellular molecules—could be effective for materializing a variety of cancer microenvironments.

Therefore, we developed cancer models according to this approach at various stages of cancer development, which could retain the individual morphology, based on different culturing methods or the number of cell types used. Since numerous studies have extensively compared 2D and 3D cancer models, our goal was to create distinct 3D in vitro cancer models, which should be helpful for the development of novel and effective therapies, but will not rely on a comparison with 2D models. Overall, we showed that the level of albumin secretion was similar in all three models, whereas collagen secretion differed between the layer and mixed models. Further, the cancer cells in the mixed model showed a more elongated cell morphology than those in the layer models, and also had distinct E-cadherin, fibronectin, and vimentin profiles, representing the EMT. Moreover, this morphological difference induced by the respective cellular environment led to different drug responses over time. Treatment with high-dose MTX on the mixed model showed higher drug resistance than the others. Thus, these results provide a solid foundation for the use of various culturing methods to effectively mimic the TME in such a way as to alter extracellular molecules, induce morphological changes, and monitor cellular drug responses.

## 4. Materials and Methods 

### 4.1. Materials

Polycaprolactone (PCL, MW = 80,000) and methotrexate (MTX, MB8407) were obtained from Sigma-Aldrich (St. Louis, MO, USA). Chloroform (99.5%) and *N*,*N*-dimethylformamide (99.5%) were purchased from Samchun Pure Chemical (Mogok-doing, Korea). Primary and secondary antibodies were obtained from Abcam (Toronto, ON, Canada). Hep G2 (HB-8065) and CCD-1112Sk (CRL-2429) cells were purchased from American Type Culture Collection (ATCC, Manassas, VA, USA). All other reagents and solutions were obtained from Sigma-Aldrich, Invitrogen (Carlsbad, CA, USA), Gibco (Rockville, MD, USA), or Thermo Fisher Scientific (Waltham, MA, USA), except where indicated otherwise.

### 4.2. Electrospinning Method to Fabricate the Polycaprolactone (PCL) Nanofibrous Membrane

The PCL nanofibrous membrane was fabricated as previously described [31]. In brief, a PCL/chloroform solution was dispensed using a syringe pump (Fusion 100, Chemyx, TX, USA) at a flow rate of 0.1 mL/h. The nozzle tip-to-distance was 100 mm, and the electrical potential between the nozzle and collector was 10 kV. To improve the interconnectivity between fibers, the nanofibrous membrane was heated at the temperature of 60 °C for 1 min and irradiated with UV lamp at 254 nm, and was then soaked in ethanol (70%) for 1 h, washed five times in phosphate-buffered saline (PBS, Gibco), and then soaked in culture medium for 24 h.

### 4.3. Characterization of the Nanofibrous Membrane

To examine the morphology, the freeze-dried nanofibrous membrane was sputter-coated with platinum for scanning electron microscopy (SEM) operated at 8–10 kV. SEM images of five randomly selected areas of each sample were analyzed using the software package Image J 1.47 to determine an average fiber diameter. The thickness was measured by using cross-sectional images of nanofibrous membrane and the porosity was analyzed using the ImageJ 1.47 with DiameterJ [32].

### 4.4. Cell Culture

Hep G2 and CCD-1112Sk cells were respectively cultured in Eagle’s minimum essential medium (ATCC, Manassas, VA, USA) and Iscove’s modified Dulbecco’s medium (ATCC, Manassas, VA, USA) supplemented with 10% fetal bovine serum (Gibco) and 1% penicillin-streptomycin (10,000 U/mL) at 37 °C in a humidified 5% CO_2_ atmosphere. Each medium was refreshed every two to three days until the cells reached 70–80% confluence for subculturing or harvesting for subsequent experiments. Eagle’s minimum essential medium was chosen for co-culture or mono culture.

The following cell seeding technique was used to develop two models of co-culture. For the layer model, 1 × 10^5^ fibroblasts were seeded onto the membrane for cell growth to become a layer that covered the entire surface of the membrane (10 × 10 mm). After three days, 1 × 10^5^ Hep G2 cells were seeded onto the fibroblast layer. For the mixture model, Hep G2 cells and fibroblasts were mixed together at a 1:1 ratio (1 × 10^5^:1 × 10^5^ cells) and seeded onto the membrane. In the mono model, 1 × 10^5^ cells were seeded onto the membrane. All cultured samples were maintained in 24-well plates in an incubator at 37 °C and 5% CO_2_ for 7 days up to a maximum of 11 days. The medium were refreshed daily, collected, and stored at −20 °C for ELISA or EIA. Figure 1b shows a brief overview of the experimental procedure with the three different co-culture models.

### 4.5. Immunocytochemistry

Immunocytochemistry was applied to evaluate cell morphology with DAPI (Invitrogen), phalloidin Alexa Fluor 488 (Invitrogen), and HNF-4 as a marker for Hep G2 cells to distinguish from CCD-1112Sk. Specific markers for epithelial and mesenchymal cells were used to analyze the differences between the layer model and mixed model. In brief, after seven days of culture, all samples were fixed in a 3.7% paraformaldehyde solution for 10 min and permeabilized with 0.2% Triton X-100 in PBS. The samples were then incubated in 1% bovine serum albumin in PBS + 0.1% Tween 20 for 30 min at room temperature to block unspecific binding of the antibodies. Rabbit anti-E-cadherin (Abcam) and rabbit anti-occludin (Abcam) were applied as the epithelial markers. The mesenchymal cell markers were rabbit anti-vimentin (Abcam, 1:100) and rabbit anti-fibronectin (Abcam, 1:200). A double-staining immunocytochemistry method was applied to label Hep G2 cells to distinguish from CCD-1112Sk. First, the primary antibody HNF-4 was added to all samples, and incubated at room temperature within 1 h. After washing with PBS three times, the secondary antibody Alexa Fluor 594 (Abcam, 1:1000) was added and further incubated for 1 h. Second, the specific primary epithelial or mesenchymal markers and the secondary antibodies Alexa Fluor 488 (Abcam, 1:1000) were applied for each individual sample according to the above protocol; the whole process was conducted in the dark. Finally, the cells were counterstained with DAPI (Sigma-Aldrich, 1:1000) to visualize the cell nucleus or phalloidin Alexa Fluor 488 alone for observing cell morphology on a fluorescent inverted microscope (Olympus IX71, Tokyo, Japan).

### 4.6. Measurement of Albumin and Collagen Type I Levels Using Enzyme-Linked Immunosorbent Assay (ELISA) and Enzyme Immunoassay (EIA)

As described above, the media were refreshed daily, collected, and stored at −20 °C during experimental periods. Human Albumin ELISA kit (ab108788, Abcam) and Procollagen Type I C-peptide (PIP) enzyme immunoassay (EIA) Kit (MK101, TAKARA BIO, Kusatsu, Japan) were used according to the manufacturer’s instructions to measure albumin and collagen type I secretions. In brief, for measurement of albumin secretion, all samples and standards were added and incubated for 3 h at room temperature and then washed five times. Tetramethybenzidine (TMB) substrate solution was then added to all wells, and the samples were incubated for 10 min at room temperature. Finally, stop solution was added, and the results were analyzed at 450 nm using a multimode microplate reader (Biotek, Winooski, VT, USA). In brief, measurement of collagen type I secretion, anti-PIP monoclonal antibodies were pre-coated onto the microplate wells. Subsequently, all samples and standards were added to each well. The results were analyzed at 450 nm using a multimode microplate reader when the color was developed with hydrogen peroxide and TMD peroxidase. The concentration of albumin and collagen type I in all samples was calculated by interpolation from standard curves.

### 4.7. Methotrexate (MTX) Drug Screening

MTX was used to evaluate the difference in drug resistance between the epithelial and mesenchymal phenotypes of Hep G2 cells. After seven days of culture, the completed growth medium was changed and replaced with medium containing different concentrations of MTX (10, 30, and 100 µM). The fresh medium containing MTX was changed after 24 h, and the maximum treatment periods were 48 h. The supernatant was collected at 24 h and 48 h for measuring albumin concentrations.

### 4.8. Statistical Analysis

At least three independent replicates were performed for each experiment. The statistic software SPSS18.0 (SPSS Inc., Chicago, IL, USA) was used for data analysis. Data are expressed as the mean ± standard deviation (SD). Statistical differences were assessed either by a non-parametric Mann-Whitney *U* test and a non-parametric one-way Kruskal-Wallis test. A value of *p* <0.05 was chosen for statistical significance.

## 5. Conclusions

This study fabricated 3D HCC models, which consist of fibroblast, HCC cells, and a nanofibrous membrane. Based on different culturing methods, three distinct models (mono, layer, and mixed models) were created and evaluated by albumin secretion, collagen secretion, observation of morphological changes, and anticancer drug resistance. From our results, we suggested that 3D cancer models should reflect the various stages of cancer progression for effective drug development.
